# Centrifugal device for dust adhesion measurement

**DOI:** 10.1016/j.ohx.2025.e00694

**Published:** 2025-09-02

**Authors:** Filip Wylęgała, Tadeusz Uhl

**Affiliations:** AGH University of Krakow, Poland

**Keywords:** Adhesion testing, Dust, Dust adhesion

## Abstract

This article details the design, construction, and operation of a benchtop centrifuge tailored for adhesion measurements. The device is intentionally simple, leveraging widely available components and exclusively employing 3D printing as the manufacturing method. The centrifuge facilitates the measurement of detachment forces, with user-adjustable sample attachment points via an interface or double-sided tape. The adhesion force is determined by considering both the detachment force and the mass of the dust; to achieve this, optical microscopy may be employed to determine the dust mass accurately. A user-friendly graphical interface allows for the input of desired rotational speeds and durations, while a built-in encoder and PID algorithm ensure precise operation.

Dust adhesion presents significant challenges for measurement, and this centrifuge addresses these challenges through a compact, modular design comprising four 3D-printed components, an Arduino Uno, a power socket, wiring, a motor controller, bearings, an encoder-equipped motor, and a plastic dome. Assembly is completed with a set of screws.

The primary application of this device is to evaluate the detachment forces of lunar regolith simulants on various materials. However, the design is versatile and can be adapted for spectrometry or as a compact centrifuge for biological applications.

## Graphical abstract

**Figure:**
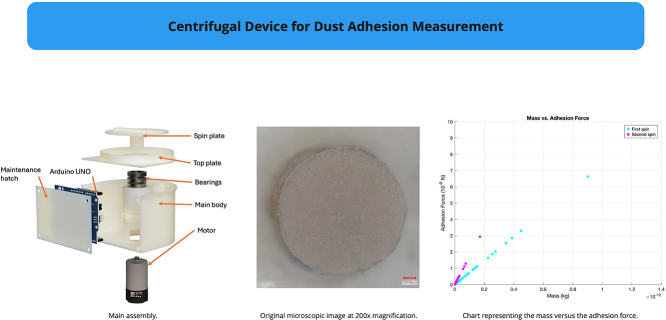

## Specifications table

**Table:** 

Hardware name	*Centrifuge for adhesion test*

Subject area	•*Engineering and material science*
Hardware type	•*Measuring physical properties and in-lab sensors*
Closest commercial analog	*No commercial analog is available*
Open source license	CC-BY-4.0
Cost of hardware	104.07 euro
Source file repository	https://doi.org/10.5281/zenodo.14841011

	

## 1 Hardware in context

Dust adhesion properties are a critical area of study in environments where fine particles pose significant challenges, such as in pharmaceutical production, food processing, and other industrial applications. A particularly important focus is on lunar regolith, which exhibits unique properties due to the Moon’s environment. Dust adhesion is a complex phenomenon driven primarily by van der Waals forces, followed by electrostatic interactions. In the presence of liquid, capillary forces also contribute to the overall adhesion behavior [1]. Lunar regolith presents unique challenges that require specialized methods for effective management. These challenges, summarized by Walton et al. [2], include vacuum conditions, rough particle morphology, and radiation. Various techniques have been developed to measure adhesion force, including those intended to simulate lunar conditions. While atomic force microscopy (AFM) can be used to measure adhesion force by modifying the tip to attach particles, it is less reliable for non-spherical particles due to approximation limitations. Petean et al. [3] suggest that centrifugal techniques, by providing statistically significant data, offer a more representative measurement of adhesion forces across an entire sample. Drop tests and kinetic methods are alternative approaches to measure adhesion; however, they often require more complex and expensive setups, as described by Zafar et al. [4]. Open-source setups for kinetic adhesion testing have also been developed by Pedrolli et al. [5]. One of the most straightforward and reliable methods for measuring dust adhesion is the use of centrifugal techniques, which are often combined with optical methods to quantify the number of particles detached from a surface, as demonstrated by Sun et al. [6]. For instance, Zhang et al. [7] employed a centrifuge operating at 52.36 rad/s, with a rotating plate diameter of 20 cm, to assess adhesion effects between dust particles and various surfaces. Barker et al. [8] explored variations of this technique, demonstrating its adaptability to different configurations, such as a rotating drum with continuous camera measurements. Oudayer et al. [9] further developed a centrifuge-based method for adhesion measurements under vacuum conditions, highlighting its utility across a range of scenarios.

Centrifugal techniques for adhesion measurement vary considerably in sample orientation, rotation plane, particle size, and rotational speed. This variability underscores the need for standardized protocols to ensure consistent data collection and to facilitate comparisons among different materials.

These techniques rely on the centrifugal force, $F_{c}$, which can be expressed as:

(1) $$F_{c}=m\cdot R\cdot \omega^{2}\;\left(\text{N}\right)$$

where:

• m is the particle mass (in kilograms, kg),
• R is the distance from the rotation axis (in meters, m),
• ω is the angular velocity (in radians per second, rad/s).

The total maximal adhesion force [9], $F_{ad}$, considering the contribution of gravitational force, can be represented as:

(2) $$F_{ad}=-\left(F_{c}+F_{g}\right)\;\left(\text{N}\right)$$

**Fig. 1:**
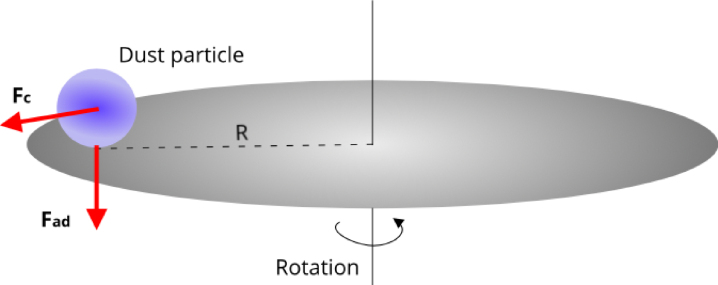
Spin plate with a particle, indicating the acting forces.

Fig. 1 illustrates the principle of the method. A spin plate rotates with a particle placed on its surface, and at the point of detachment, the adhesion force is balanced by the sum of the centrifugal and gravitational forces.

## 2 Hardware description

One of the key advantages of this design is its simplicity and accessibility, offering a unique approach to adhesion measurement. In comparison, the open-source technique developed by Pedrolli et al. [5] costs approximately 1300 EUR. By contrast, the proposed centrifugal method is both less complex and significantly more cost-effective.

### 2.1 Main body and mechanical components

**Fig. 2:**
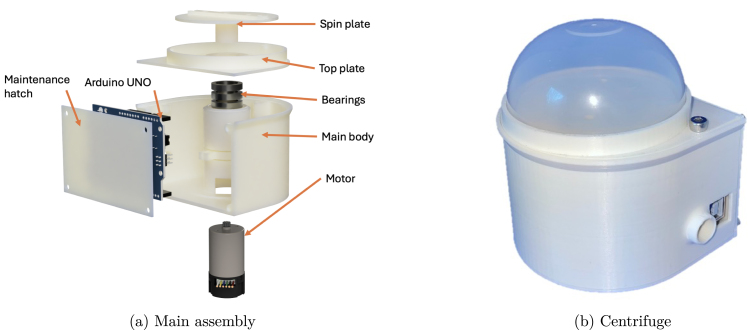
Comparison of main assembly and assembled centrifuge.

The main body consists of three 3D-printed components that can be fabricated using entry-level materials such as PLA, PETG, or ABS. The complete mock-up is presented in Fig. 2(a). The parts are assembled using screws. While the threads are modeled directly into the printed parts, reinforcement using helicoils can be implemented if required to enhance durability.

Inside the body, there is a socket designed to accommodate the motor and three bearings. The enclosure includes pre-defined openings for power connections and USB communication with an Arduino Uno. A dedicated space for the Arduino is provided on the maintenance hatch. The motor controller is loosely placed, allowing flexibility in using different approaches such as soldered or breadboard configurations. The design does not impose limitations on these choices.

The assembled centrifuge is shown in Fig. 2(b). The design emphasizes accessibility and ease of assembly, making it suitable for a wide range of users with varying levels of expertise.

### 2.2 Spin plate

The fourth 3D printed component is the spin plate, which features a single attachment point and two holes for sample adapters. Since samples can vary significantly, designing a universal adapter is challenging. As a result, researchers may opt for specific adapters or use double-sided tape to mount the samples securely onto the plate. The motor connects to the spin shaft via a keyed connection, ensuring stability and efficient power transfer during operation. Given the dusty nature of the setup, a protective dome has been employed to prevent contamination of the laboratory environment. The semi transparent dome, from a food container, offers a cost-effective and accessible solution. It is mounted using a simple twist-and-pull technique.

The device can be customized for a wide range of applications in biology and chemistry, including spin coating and other centrifugal processes, by designing appropriate adapters. The primary tasks include:

• Characterizing surface energy parameters.
• Comparing materials based on their adhesion properties.

### 2.3 Electronics

**Fig. 3:**
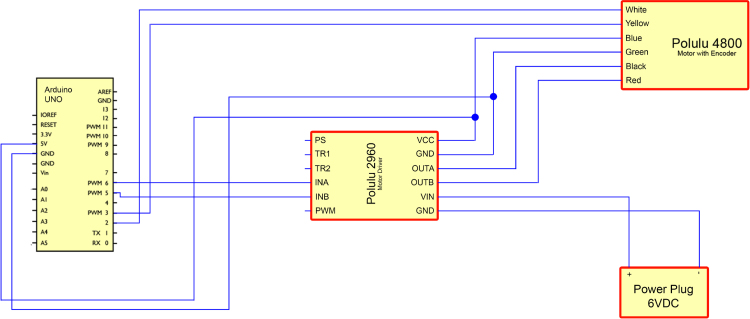
Electronics diagram.

The Arduino Uno was chosen as the controller for the setup due to its accessibility and ease of programming. It is connected to a BD65496MUV brushed motor controller, which drives the motor. The motor is powered by an external 6 V power supply, connected to a socket mounted in the main body adjacent to the Arduino’s USB port. The electronic schematic is shown in Fig. 3.

The chosen motor for this design is the Pololu 4800, which includes a 48 CPR (counts per revolution) encoder. It is connected to Arduino to provide real-time information about the shaft rotation, enabling precise control and monitoring. The version without a gearbox was selected to achieve high rotational speeds. The motor’s maximum rated speed is 10,000 rpm under no-load conditions. However, the actual maximum operational speed of the design may vary depending on factors such as print quality, residual torque in the bearings, and other mechanical tolerances.

### 2.4 Software

**Fig. 4:**
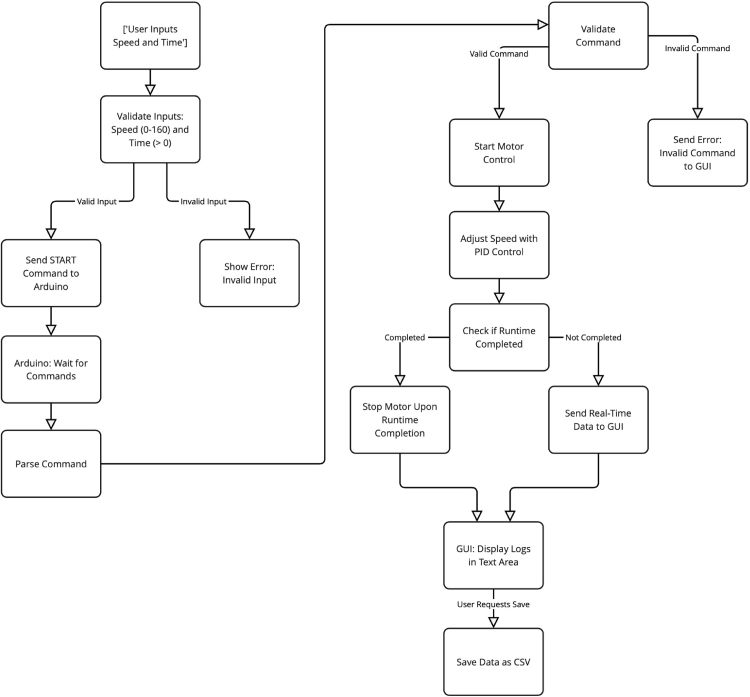
Software block diagram.

Custom software was developed for both the Arduino and Python environments to control the motor. The Python-based graphical user interface (GUI) enables users to configure speed runs for specified durations, while simultaneously recording and saving speed (in RPM) and runtime data for subsequent analysis. The working principle of the software is presented in Fig. 4.

## 3 Design files summary

All the files are made available on online repository in editable and ready to use files. All the files are shared under Creative Commons Attribution-ShareAlike 4.0 International License. The files links are presented in Table 1

**Table 1:** Summary of design files.

Design filename	File type	Open source license	Location of the file
centrifuge.3mf	3D model file	CC-BY-4.0	Doi:10.5281/zenodo.14841011
Dust segmentation.mlx	MATLAB Live Script	CC-BY-4.0	Doi:10.5281/zenodo.14841011
Main body.stl	CAD file (3D model)	CC-BY-4.0	Doi:10.5281/zenodo.14841011
Maintenance hatch.stl	CAD file (3D model)	CC-BY-4.0	Doi:10.5281/zenodo.14841011
motor_opt.ino	Arduino code	CC-BY-4.0	Doi:10.5281/zenodo.14841011
motor_opt.py	Python script	CC-BY-4.0	Doi:10.5281/zenodo.14841011
README.txt	Documentation	CC-BY-4.0	Doi:10.5281/zenodo.14841011
Spin plate.f3d	CAD file (Fusion 360 model)	CC-BY-4.0	Doi:10.5281/zenodo.14841011
Spin plate.stl	CAD file (3D model)	CC-BY-4.0	Doi:10.5281/zenodo.14841011
Top plate.stl	CAD file (3D model)	CC-BY-4.0	Doi:10.5281/zenodo.14841011

			

## 3D designs

Ready-to-use 3D printing files are provided in the .stl format, which can be directly imported into slicing software for immediate printing. To further streamline the printing process, a printing profile is included in the .3mf format, ensuring compatibility with Bambu Lab Studio. Additionally, for the Spin Plate, an .f3d file is included to facilitate easier adjustments to the model.

## Software code

The software includes several essential components designed to facilitate operation and data processing. The Arduino code, provided in the .ino format, is ready for uploading to Arduino-compatible microcontrollers to control the hardware. A Python script is included in the .py format, offering tools for executing additional processes and simulations. To assist users in setting up the environment and understanding the system, a detailed README file is available in .txt format. Furthermore, a MATLAB script for dust segmentation is provided in the .mlx format, enabling robust data analysis and visualization.

## 4 Bill of materials summary

The bill of materials consists of the following elements, as presented in Table 2.

**Table 2:** Bill of materials.

Designator	Component	No.	Unit Cost (€)	Total (€)	Source	Material
Microcontroller	Arduino Uno	1	27.90	27.90	https://botland.store/arduino-basic-boards/1060-arduino-uno-rev3-module-a000066-7630049200050.html	Non-specific

Motor	Pololu 4800	1	30.54	30.54	https://www.pololu.com/product/4800	Non-specific
Controller	Pololu 2960	1	11.59	11.59	https://www.pololu.com/product/2960	Non-specific

Power plug	Power plug	1	1.9	1.9	https://botland.store/wires-and-power-connectors/413-dc-plug-55x25mm-for-cable-10pcs-5904422355531.html	Non-specific

Power socket	Power Socket	1	2.50	2.50	https://botland.store/wires-and-power-connectors/9353-dc-55-x-25mm-socket-for-case-12mm-10pcs-5904422338039.html	Non-specific

Bearings	Bearings	3	2.65	7.95	https://www.kugellager-express.de/	Metal

Dome	Food container	1	0.99	0.99	https://www.ikea.com/at/en/p/splitterny-snack-container-transparent-light-grey-brown-30586293/	Polymer

Breadboard	Breadboard	1	0.90	0.90	https://botland.store/breadoards/605-breadboard-170-holes-white-5904422372866.html	Polymer

Cables Set	Cables Set	1	4.00	4.00	https://botland.store/various-wires/19946-connecting-cables-set-justpi-20cm-3-x-40pcs-m-m-f-f-m-f-120pcs-5904422328702.html	Non-specific

Fasteners	Fasteners Set	1	2.90	2.90	https://botland.store/screws-and-nuts/637-screws-nuts-and-washers-set-330pcs-5410329304478.html	Metal

Filament	Filament Devil Design PLA 1.75 mm 0.33 kg	1	12.90	12.90	https://botland.store/pla-filaments/24978-filament-devil-design-pla-175mm-033~kg-marble-light-5902280032199.html	Polymer

### Equipment required for assembly and operation

• **3D Printer:** A Bambulab X1C printer was used with ASA filament.
• **Screwdriver:** For securing hardware components.
• **Soldering Iron:** Necessary if permanent connections of wires are required.
• **Laboratory Power Supply :** Necessary to power the system.
• **Cyanoacrylate Adhesive:** Used if permanent bonding of hardware components is needed.

### Software utilized

• **3D Printing Preparation:** Files were prepared using *Bambulab Studio*.
• **3D Modeling:** All models were designed in *Autodesk Fusion 360*.
• **Programming:** Code was developed using:
  – *Visual Studio Code* for Python scripting.
  – *Arduino IDE* for controlling the Arduino.
  – *Matlab* for image processing.

## 5 Build instructions

### 5.1 Printing

**Fig. 5:**
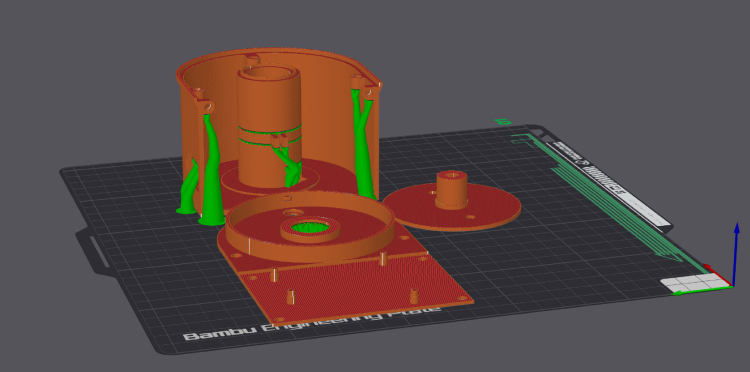
Sliced 3D models in Bambu Lab Studio.

The printing profile is provided in the repository, and all parts can be fabricated using the same settings. Fig. 5 shows the sliced 3D model in Bambu Lab Studio. After printing, carefully remove any supports and inspect the quality of the prints for potential defects or inconsistencies.

In case of 3D printing use the following rules:

• Use supports to ensure proper overhang stability during printing.
• Set the layer height to 0.16 mm or less for accurate representation of threads and fine details.
• Conduct a fit check after printing to verify compatibility and alignment between contacting elements.

### 5.2 Electronics assembly

**Fig. 6:**
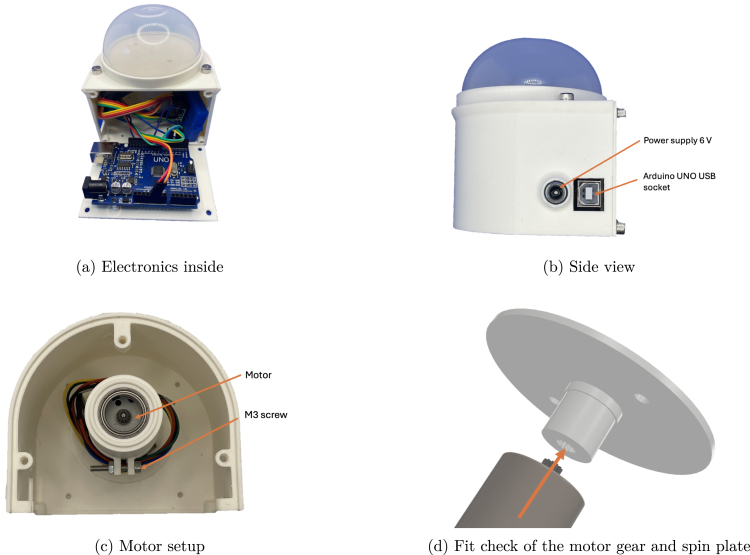
Overview of the components: electronics, side view, motor setup, and fit check.

The following steps outline the procedure for assembling the electronic components:

1. Perform the fit check between the spin plate and the motor (Fig. 6(d)). This interface is critical to ensure proper alignment and functionality.
2. Insert the motor into the motor socket (Fig. 6(c)) and secure it using an M3 screw with a nut. Ensure the motor is fully seated to avoid misalignment during operation.
3. Place the Arduino board on the maintenance hatch (Fig. 6(a)), aligning its pins with the corresponding holes. For a more permanent installation, adhesive (e.g., glue) may be used to secure the board.
4. Mount the motor controller onto the breadboard, ensuring stable connections.
5. Install the power socket into its designated hole, securing it with adhesive (Fig. 6(b)). Alternatively, two wires may be routed through the hole for external connections.

### 5.3 Wiring instructions

**Fig. 7:**
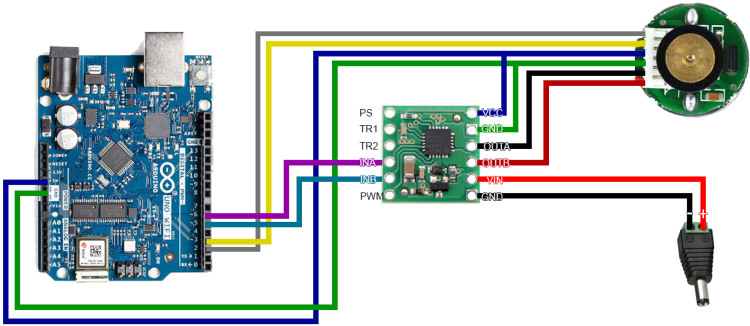
Wiring diagram.

Connect the wires according to the provided schematic shown in Fig. 7. For a durable and long-lasting setup, solder the wires instead of using breadboard connections.

**Pololu 4800 (Motor with Encoder):**

• **White cable:** Connect to Pin 2 of Arduino Uno.
• **Yellow cable:** Connect to Pin 3 of Arduino Uno.
• **Blue cable:** Connect to VCC on the motor driver.
• **Green cable:** Connect to GND on the motor driver.
• **Black cable:** Connect to OUTA on the motor driver.
• **Red cable:** Connect to OUTB on the motor driver.

**Power supply:**

• **Red cable (+):** Connect to VIN on the motor driver.
• **Black cable** (−): Connect to GND on the motor driver.

**Pololu 2960 (Motor Driver):**

• **Pin INA (Purple):** Connect to Pin 6 of Arduino Uno.
• **Pin INB (Blue):** Connect to Pin 5 of Arduino Uno.
• **Pin VCC (Dark Blue):** Connect to 5 V on Arduino Uno.
• **Pin GND (Green):** Connect to GND on Arduino Uno.

## 6 Operation instructions

The repository includes a ‘README.md‘ file that provides detailed instructions for setting up and running the Python and Arduino environments. Ensure the Arduino is connected, and the motor is powered before operating the system.

**Fig. 8:**
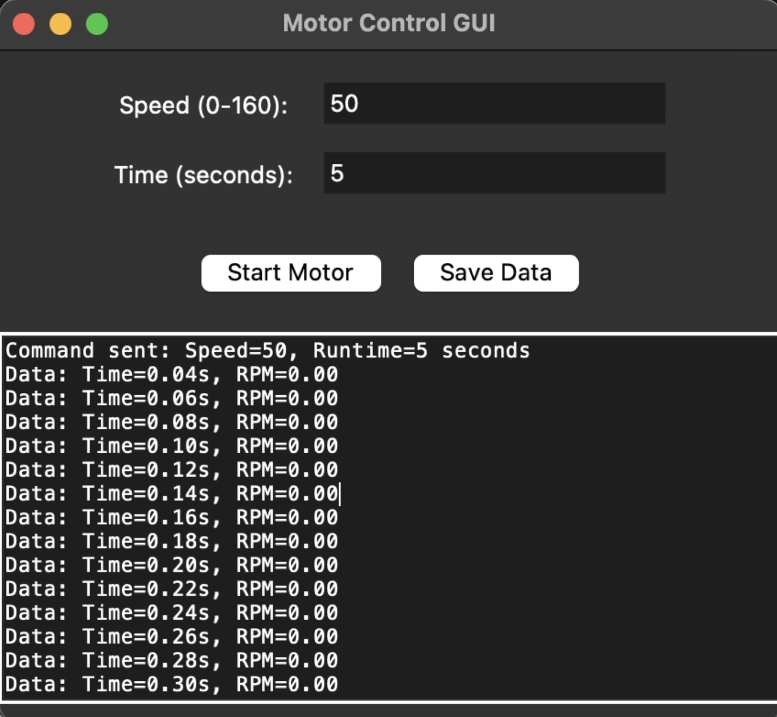
View of the motor GUI.

The graphical user interface (GUI) visible in Fig. 8 provides an intuitive way to control and monitor the motor.

For controlling follow the steps:

1. **Input Speed-Time Commands:** Enter the desired speed (RPM) and runtime (seconds) as comma-separated pairs in the text box, one pair per line (e.g., 30, 5). Multiple commands will execute sequentially.
2. **Start Motor:** Click the **Start Motor** button to execute the commands. The motor will adjust its speed based on the specified values.
3. **Monitor and Save Data:** Real-time RPM and time data will display in the log window. Click the **Save Data** button to export the data as a CSV file.

**Note: Always use the protective dome when spinning the rotor. During the first run, start with a slow RPM to verify the structural integrity of the setup.**

## 7 Validation and characterization

**Fig. 9:**
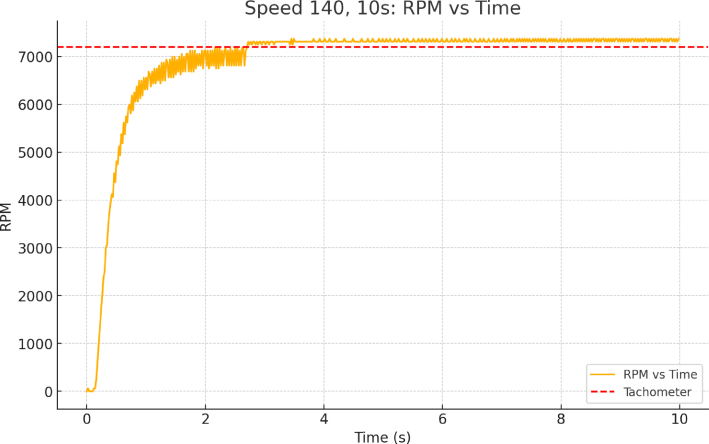
Speed vs. time chart with labeled measured levels.

Both the speed control tests and the initial characterization of dust behavior were performed to validate the system. The rotational speed was measured using a digital tachometer (Uni-T UT373). A piece of reflective tape was affixed to the rotating plate, and the tachometer’s laser was directed toward the tape. The measured speed provided a verification of the encoder’s readings. However, the Uni-T UT373 exhibits significant limitations: its low refresh rate and lack of capability for continuous speed recording restrict its use to a rough verification of the magnitude of the encoder’s measurements. Fig. 9 displays the speed versus time chart with the measured levels labeled.

It is important that the velocity remains constant and does not exhibit significant fluctuations. The provided ramp is adequate for the experimental setup. For subsequent calculations, the maximum attained velocity should be used to determine the detachment force.

## 8 Dust characterization

The centrifuge’s functionality was evaluated using the Lunar Regolith Simulant LX-M100, manufactured by Lunex Technologies. Initially, the simulant was sieved through a 100 μm mesh followed by a 50 μm sieve, and a uniform layer of dust was deposited onto the sample plates.

Dust application via a sieve resulted in a uniform layer of dust being deposited onto the sample plate. The plates were then inverted to remove loosely adhered particles, ensuring that only dust in direct contact with the surface remained. The prepared samples were subsequently taped to the spinning plate using double-sided adhesive tape and subjected to the designated rotation speeds.

The experimental procedure involved spinning the samples at two distinct speeds-6800 RPM and 10,400 RPM-for a duration of 10 s per spin. Two different samples were prepared named A and B.

For imaging and analysis, a Keyence VHX-7000 microscope with a 200× magnification, vertical stacking, and horizontal stitching capabilities was employed. This configuration ensured that the entire 2.5 mm Polyether Ether Ketone (PEEK) sample was captured in detail. The dust, which had been pre-sieved with a 50 μm sieve; the majority of its particles are sub-micrometer in size.

**Fig. 10:**
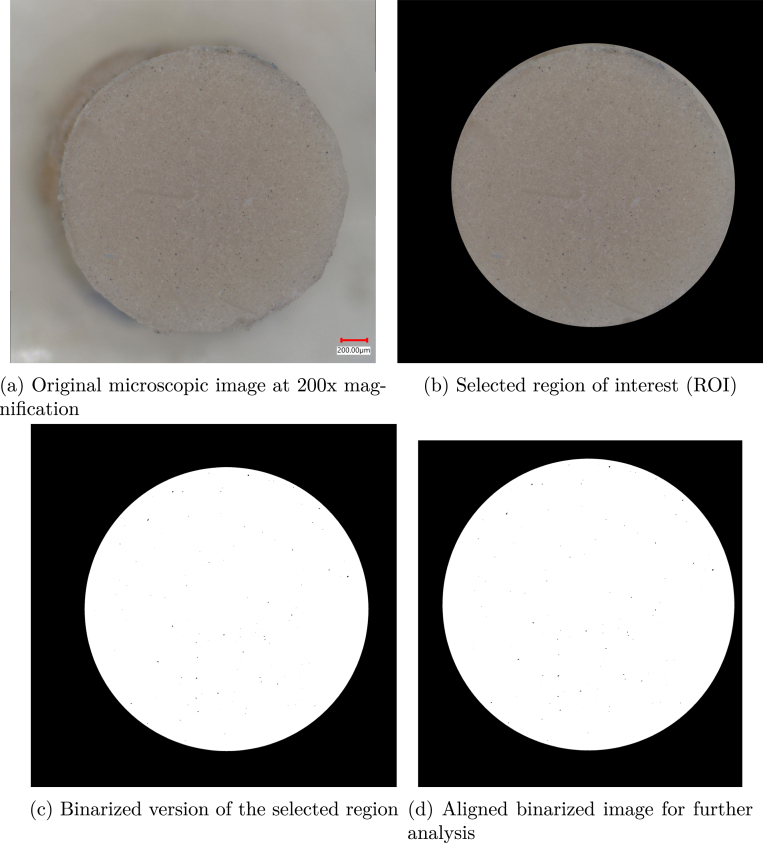
Image processing stages.

Post-processing was performed using MATLAB 2024B, as illustrated in Fig. 10. The workflow involved loading the images, computing the pixel-to-micrometer conversion factor (0.7041 μm/pixel), and selecting a consistent region of interest (ROI) across all images. Subsequently, thresholding was applied to differentiate dust particles from the background.

**Fig. 11:**
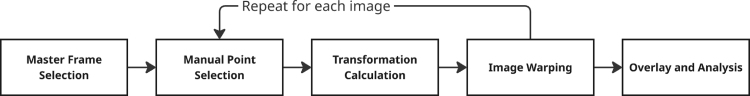
Alignment and segmentation workflow repeated for each image set.

The alignment procedure applied to each image set is illustrated in Fig. 11. For every image, the workflow begins with selecting a master frame-typically the last image in the sequence due to its minimal particle count. Next, three consistent reference points are manually identified on both masks to guide the alignment. A similarity transformation is then calculated using fitgeotrans in MATLAB, which is applied to the pre-spin image via imwarp for spatial alignment.

**Fig. 12:**
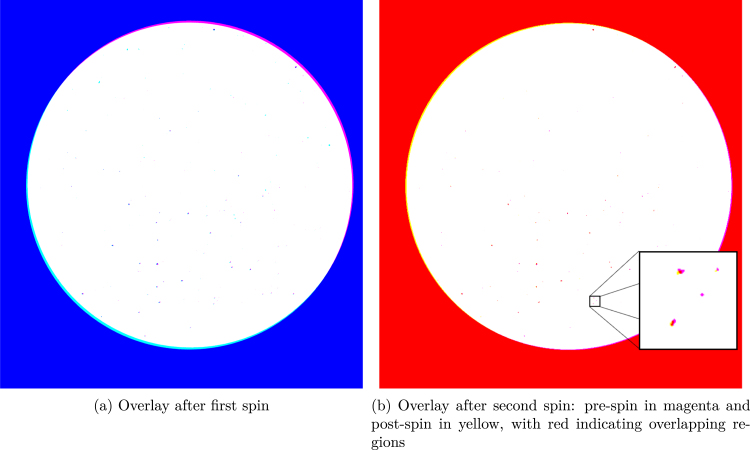
Overlay images after successive spins arranged in a row. (For interpretation of the references to color in this figure legend, the reader is referred to the web version of this article.)

After alignment, the images were overlaid using distinct colors to highlight the changes induced by each spin. For the first spin, the pre-spin image is rendered in cyan, while the post-spin image is displayed in magenta. In the resulting overlay, dark blue regions indicate the cyan particles that were removed during the first spin. A slight misalignment is observable in both cases. Analogously, for the second spin, the pre-spin image is shown in magenta and the post-spin image in yellow, which results in red overlapping regions. In this overlay, the magenta areas correspond to particles that detached during spinning. The process is illustrated in Fig. 12.

After extracting the images of each particle, the area was determined from the segmented images. Using the known aspect ratio (0.656), the height of each particle was estimated as:

(3) $$h=\sqrt{\text{Area}}\times \text{Aspect Ratio}\;\left(\text{m}\right)$$

With the area and estimated height, the volume was approximated as:

(4) $$V=\text{Area}\times h\;\left({\text{m}}^{3}\right)$$

Knowing the solid density of the particles ($2.96\;{\text{g/cm}}^{3}$), the mass was calculated by converting the volume into cubic meters and applying:

(5) m=V×ρ(kg)

where ρ is the density in kg/m${}^{3}$.

The radius of rotation was 0.014455 m. Using the calculated mass and rotational parameters, the centrifugal force that could be understood as the particle detachment force for each particle was determined as:

(6) $$F_{c}=m\cdot \omega^{2}\cdot r\;\left(\text{N}\right)$$

where ω is the angular velocity (in rad/s) calculated from the RPM values (6800 RPM for cyan, 10400 RPM for magenta).

The adhesion force acting on the particles was determined as the sum of the detachment force ($F_{c}$) and the gravitational force ($F_{g}$), where $F_{g}=m\cdot g$, and g represents the acceleration due to gravity ($9.81\;{\text{m/s}}^{2}$). This adhesion force, expressed as:

(7) $$F_{\text{ad}}=-\left(F_{c}+F_{g}\right)\;\left(\text{N}\right),$$

The terramechanical properties of the regolith simulant were presented in Table 3

**Table 3:** Physical and terramechanical properties.

Property	Value
Solids density (ρ)	2.96 g/cm^3^
Bulk density ($\rho_{B}$)	1.41 g/cm^3^
Tapped density ($\rho_{T}$)	1.81 g/cm^3^
Hausner ratio (HR)	1.28
Angle of repose (A)	41.9°–45.8°
Average aspect ratio	>0.656
Particle size range	0–2.0 mm
Median particle size	87 μm

### 8.1 Results

**Fig. 13:**
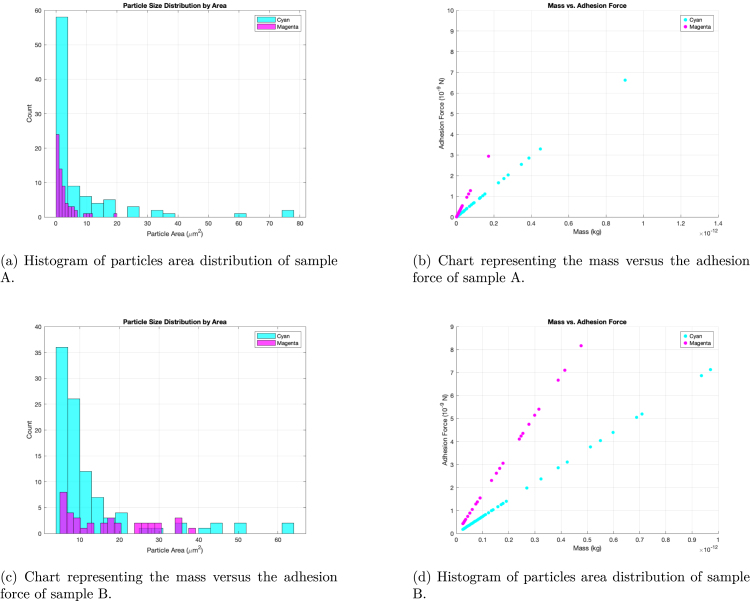
Results of the test for sample A and B. (For interpretation of the references to color in this figure legend, the reader is referred to the web version of this article.)

The analyzed particles are presented in Fig. 13, Fig. 13. In both histograms, the cyan color represents the particles detached during the first spin, while the magenta indicates those detached during the second spin. Fig. 13, Fig. 13 present the adhesion force versus mass for both samples. In these plots, each point corresponds to a detached particle, with color indicating the spin cycle. The adhesion force, calculated as the sum of centrifugal and gravitational components, represents the maximum detachment force for each particle.

### 8.2 Conclusions and discussion

It is possible to test adhesion using the device presented in this study. The lunar dust simulant exhibits considerable variability in both shape and color, which makes both the image processing and the adhesion measurement challenging. The centrifugal forces applied during the experiments serve as a proxy for the detachment force, representing the maximum force by which a particle adheres to the substrate. Although automatic image alignment improves the quality of the analysis, variations in particle shape, brightness, and lighting conditions introduce additional complexity.

The mass obtained from the study was calculated as an approximate value due to the irregular nature of the material; furthermore, there is no prior work specifically addressing this simulant in combination with PEEK substrates. It is also important to note that the measured adhesion force for any given particle corresponds to the maximum value, while individual particles may exhibit lower adhesion forces. Consequently, force measurements should be performed over multiple spins to ensure statistically robust results. In the initial spin, a large number of small particles detached, likely because they possessed lower adhesion forces. However, the centrifugal force was sufficiently high to detach not only these particles but also larger aggregates. The magnitude of the calculated adhesion force (on the order of nanonewtons) is similar to that reported by Oudayer et al. [9], despite differences in substrate and vacuum conditions, and also aligns with results from Zhang et al. [7], who performed their study under atmospheric pressure.

Samples A and B differ both in particle size distribution and in the resulting adhesion force profiles. This is evident from the broader distribution in Sample A and the overall higher forces observed in Sample B. These variations are expected, as in real-world applications the distribution and coverage of particles will rarely be uniform. Notably, Sample A had fewer particles deposited, which contributes to the generally lower adhesion forces observed in that case.

This variation in particle count and size is not a limitation but a feature of realistic testing scenarios. The primary purpose of this device is to serve as a comparative tool-allowing rapid assessment of adhesion characteristics across different materials or surface treatments. In this context, absolute precision is less important than the ability to distinguish trends and identify materials with lower adhesion tendencies.

The device is expected to perform optimally with particles of uniform size, such as spheres. Therefore, an optimal strategy for investigating adhesion forces would involve preparing several samples with the same surface characteristics, positioned at various distances from the center of rotation. Nonetheless, even in its current configuration, the system is effective for screening and comparative evaluation of material–dust interactions.

### 8.3 Capabilities and limitations

• The maximum and minimum rotational speeds depend on factors such as bearing resistance, print quality, and mechanical tolerances of the 3D-printed components.
• RPM values cannot be directly entered into the system. Instead, the motor input must be calibrated experimentally to determine the corresponding rotational speed. This approach compensates for system-specific variability.
• The device is capable of reaching high rotational speeds, and the integrated PID controller ensures stable operation after an initial ramp-up period.
• Its compact design allows the system to be conveniently positioned under optical instruments, including microscopes, for pre- and post-test imaging.
• The sample mounting interface supports both fixed and customizable attachments, allowing flexibility in testing different substrate geometries without requiring permanent fixtures.
• Adhesion force is estimated indirectly, based on calculated mass and angular velocity. As such, accuracy depends on segmentation quality and physical assumptions (e.g., particle density and shape).
• The most effective use case is comparative testing. Multiple samples can be placed simultaneously at equal radial distances, enabling direct comparison of powder adhesion characteristics across different materials within a single test run.

## CRediT authorship contribution statement

**Filip Wylęgała:** Writing – original draft, Software, Methodology, Conceptualization. **Tadeusz Uhl:** Supervision, Funding acquisition, Conceptualization.

## Declaration of Generative AI and AI-assisted technologies in the writing process

During the preparation of this work, the author(s) used DeepL and ChatGPT to enhance readability of the text and code clarity. After using these services, the author(s) reviewed and edited the content as necessary and take full responsibility for the content of the publication.

## Funding

This work was supported by IDUB AGH: University grant system for research projects carried out with the participation of doctoral students [grant numbers 10445, 2024]

## Declaration of competing interest

The authors declare that they have no known competing financial interests or personal relationships that could have appeared to influence the work reported in this paper.
